# An *in vitro* model of murine middle ear epithelium

**DOI:** 10.1242/dmm.026658

**Published:** 2016-11-01

**Authors:** Apoorva Mulay, Khondoker M. Akram, Debbie Williams, Hannah Armes, Catherine Russell, Derek Hood, Stuart Armstrong, James P. Stewart, Steve D. M. Brown, Lynne Bingle, Colin D. Bingle

**Affiliations:** 1 Academic Unit of Respiratory Medicine, Department of Infection, Immunity and Cardiovascular Disease, University of Sheffield, Sheffield S10 2JF, UK; 2 MRC Mammalian Genetics Unit, Harwell OX11 0RD, UK; 3 Oral and Maxillofacial Pathology, Department of Clinical Dentistry, University of Sheffield, Sheffield S10 2TA, UK; 4 Department of Infection Biology, Institute of Infection and Global Health, University of Liverpool, Liverpool L3 5RF, UK

**Keywords:** Otitis media, Middle ear epithelium, *Bpifa1*, Air–liquid interface, NTHi

## Abstract

Otitis media (OM), or middle ear inflammation, is the most common paediatric disease and leads to significant morbidity. Although understanding of underlying disease mechanisms is hampered by complex pathophysiology it is clear that epithelial abnormalities underpin the disease. There is currently a lack of a well-characterised *in vitro* model of the middle ear (ME) epithelium that replicates the complex cellular composition of the middle ear. Here, we report the development of a novel *in vitro* model of mouse middle ear epithelial cells (mMECs) at an air–liquid interface (ALI) that recapitulates the characteristics of the native murine ME epithelium. We demonstrate that mMECs undergo differentiation into the varied cell populations seen within the native middle ear. Proteomic analysis confirmed that the cultures secrete a multitude of innate defence proteins from their apical surface. We showed that the mMECs supported the growth of the otopathogen, nontypeable *Haemophilus influenzae* (NTHi), suggesting that the model can be successfully utilised to study host–pathogen interactions in the middle ear. Overall, our mMEC culture system can help to better understand the cell biology of the middle ear and improve our understanding of the pathophysiology of OM. The model also has the potential to serve as a platform for validation of treatments designed to reverse aspects of epithelial remodelling that underpin OM development.

## Abstract (precis)

**Summary:** Development and systematic characterisation of an *in vitro* otopathogenic infection model of the murine middle ear epithelium as a tool to better understand the complex pathophysiology of Otitis media.

## INTRODUCTION

Otitis media (OM), or inflammation of the middle ear, is the most prevalent childhood disease, a leading cause of surgery in developed countries, and a significant reason for paediatric mortality in developing countries. Eighty percent of children suffer from at least one episode of OM by three years of age (Bakaletz, 2010; Woodfield and Dugdale, 2008).

The middle ear epithelium is similar to the respiratory epithelium and is composed of ciliated cells, secretory cells, non secretory cells and basal cells. Secretory cells are responsible for the production of mucins and various anti-microbial proteins such as lactotransferrin, lysozyme, defensins and surfactants (Lim et al*.*, 2000; McGuire, 2002). The epithelium, along with its secretions, is involved in maintaining homeostasis and sterility within the middle ear cavity (MEC). Epithelial remodelling, characterised by mucociliary metaplasia and infiltration of the MEC with inflammatory cells, is a common feature of OM (Straetemans et al*.*, 2001).

In most animals, the middle ear is a relatively inaccessible organ lined by a thin mucociliary epithelium and sampling of the mucosa is a terminal procedure. Human middle ear tissue can be acquired only during surgical procedures and this limits the amount of sample available for study of OM. Culturing of middle ear cells *in vitro* enables maximisation of the available material, allows the effect of modifying culture conditions to be studied more easily and also allows functional studies to be performed. Previously, attempts have been made to culture middle ear epithelial cells from a number of organisms including rats (Toyama et al*.*, 2004; Ueyama et al*.*, 2001; Van Blitterswijk et al*.*, 1986), mice (Tsuchiya et al*.*, 2005), chinchillas (Amesara et al*.*, 1992; Nakamura et al*.*, 1991), gerbils (Herman et al*.*, 1992; Portier et al*.*, 2005; Takeno, 1990), rabbits (Schousboe et al*.*, 1995) and humans (Choi et al*.*, 2002; Chun et al*.*, 2002; Moon et al*.*, 2000). These studies have included organ and explant cultures, primary cell cultures and development of middle ear cell lines. However, there remains a lack of a robust *in vitro* middle ear epithelial model that differentiates into the different epithelial cell types of the middle ear and is free of fibroblast contamination. This has greatly restricted the ability to identify the function of different cell types and their products within the middle ear and limits our understanding of the pathophysiology of OM development.

We report here the development of a novel *in vitro* primary model of the mouse middle ear epithelium using air–liquid interface (ALI) culture and systematically characterise the different cell types present in the middle ear. We also demonstrate that this culture system can be utilised to study host–pathogen interactions within the middle ear and thus has the potential to allow investigation of the mechanisms of OM pathogenesis.

## RESULTS

**Fig. 1.**
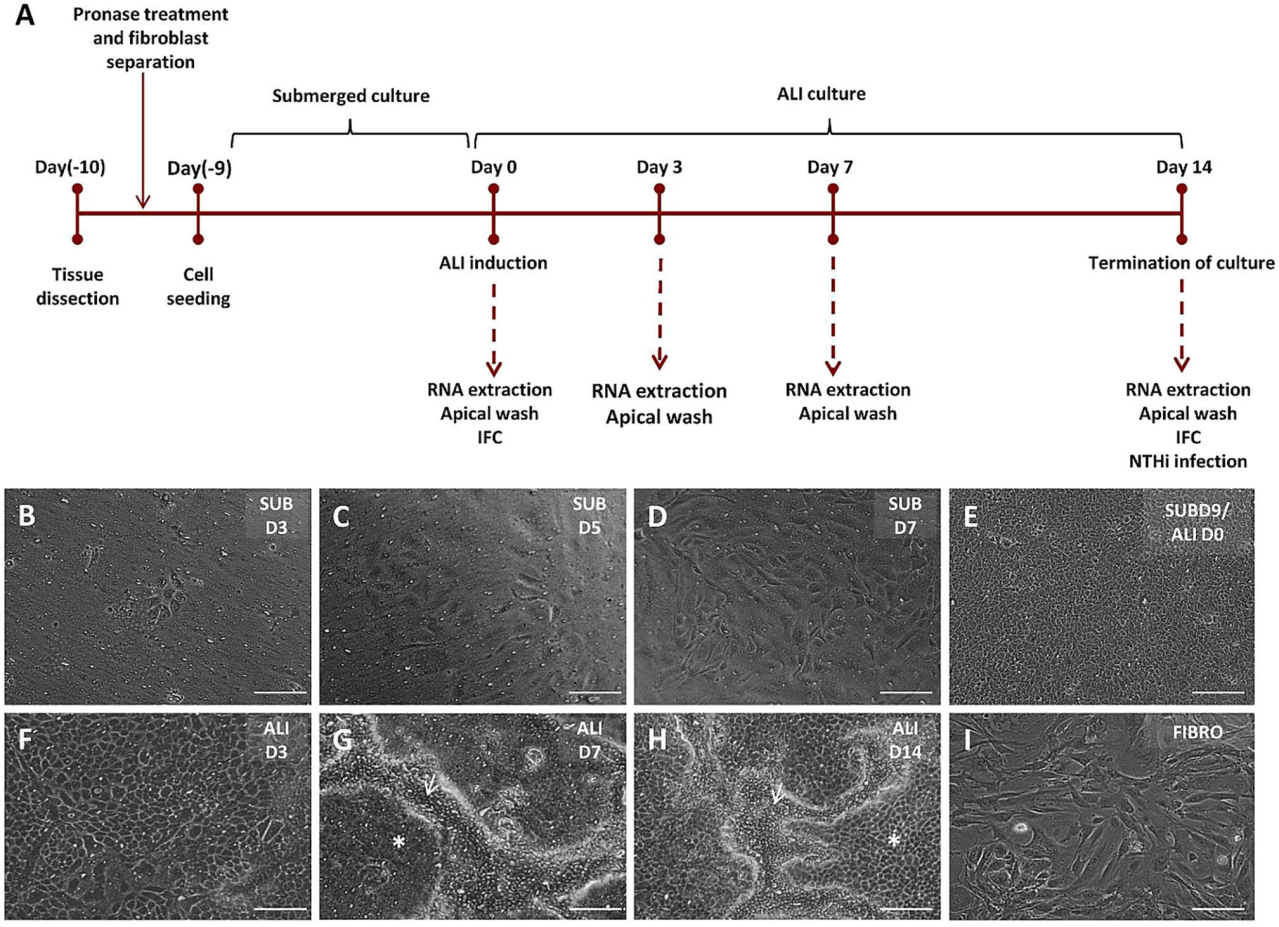
**Primary culture of mouse middle ear epithelial cells.** (A) Timeline for culture of mMECs. Bullae were dissected, treated with pronase for dissociation of the middle ear epithelial cells and fibroblasts were excluded from culture by differential adherence to plastic. Epithelial cells were grown in submerged culture until confluence, before ALI was induced. Samples for transcriptional and proteomic analysis were collected at regular time points. (B-I) Phase-contrast images showing cells in culture under 10× magnification. Under the proliferative submerged conditions (SUB), a small number of cells attached to form epithelial islands 3 days after seeding (B). The cells proliferated faster from day (D)5 (C) through day 7 (D) and formed a confluent monolayer at day 9. This was termed ALI day 0 (E). Morphology of cells changed from ALI day 3 (F) and clusters of compactly arranged cells started forming at ALI day 7 (G). (H) ALI day 14 cultures were composed of flat polygonal and compactly clustered pseudostratified cells with active cilia. White arrows mark elevated ciliated cells and asterisks mark flatter polygonal cells. (I) Fibroblasts cultured on plastic plates through differential adhesion method. Scale bars: 200 μm.

We established an air–liquid interface (ALI) culture system to model the mouse middle ear epithelium *in vitro* (Fig. 1A). We performed a morphological analysis and systematically characterised the various epithelial cell types expressed by our model in comparison with the native mouse middle ear epithelium.

### Cell culture characteristics

The average number of epithelial cells isolated was 74,667±10,621 (mean±s.e.m.) cells per MEC (*n*=12 batches). Primary culture of mMECs proceeded in two phases – a proliferative phase in submerged culture and a differentiation phase at an air–liquid interface (Fig. 1B-H). mMECs were seeded at a density of 1×10^4^ cells/membrane and 3 days after seeding 16.8±2.6% adhered to the membrane and had started forming small epithelial islands (Fig. 1B). The attached cells began to elongate to establish contact with neighbouring cells and began to proliferate rapidly from day 5 to day 7 (Fig. 1C,D). Cells formed a confluent monolayer of flat, polygonal cells within 9 to 10 days in submerged culture (ALI day 0) in the presence of a selective Rho kinase inhibitor (hereafter ROCKi) (Fig. 1E). The morphology of the cells became more complex when transferred to ALI. At ALI day 3, cells started changing in size and shape (Fig. 1F) and by ALI day 7 two distinct sub-populations of cells could be observed, the majority of which were flat polygonal cells, intersected with clusters of slightly elevated, more compactly arranged cells (Fig. 1G). Around ALI day 9, ciliary beating could be seen in these clusters under phase-contrast microscope. ALI day 14 cells (1.2×10^5^ cells/membrane) displayed a cobble-stone appearance with well-defined cell boundaries, and were a combination of flat polygonal cells and compactly clustered elevated cells with actively beating ciliated cells (Fig. 1H; Movie 1). No cells with fibroblast-like morphology were seen in the mMEC ALI cultures. However, fibroblasts were isolated through differential adherence to plastic during the cell isolation process (Fig. 1I).

### Cell morphology

**Fig. 2.**
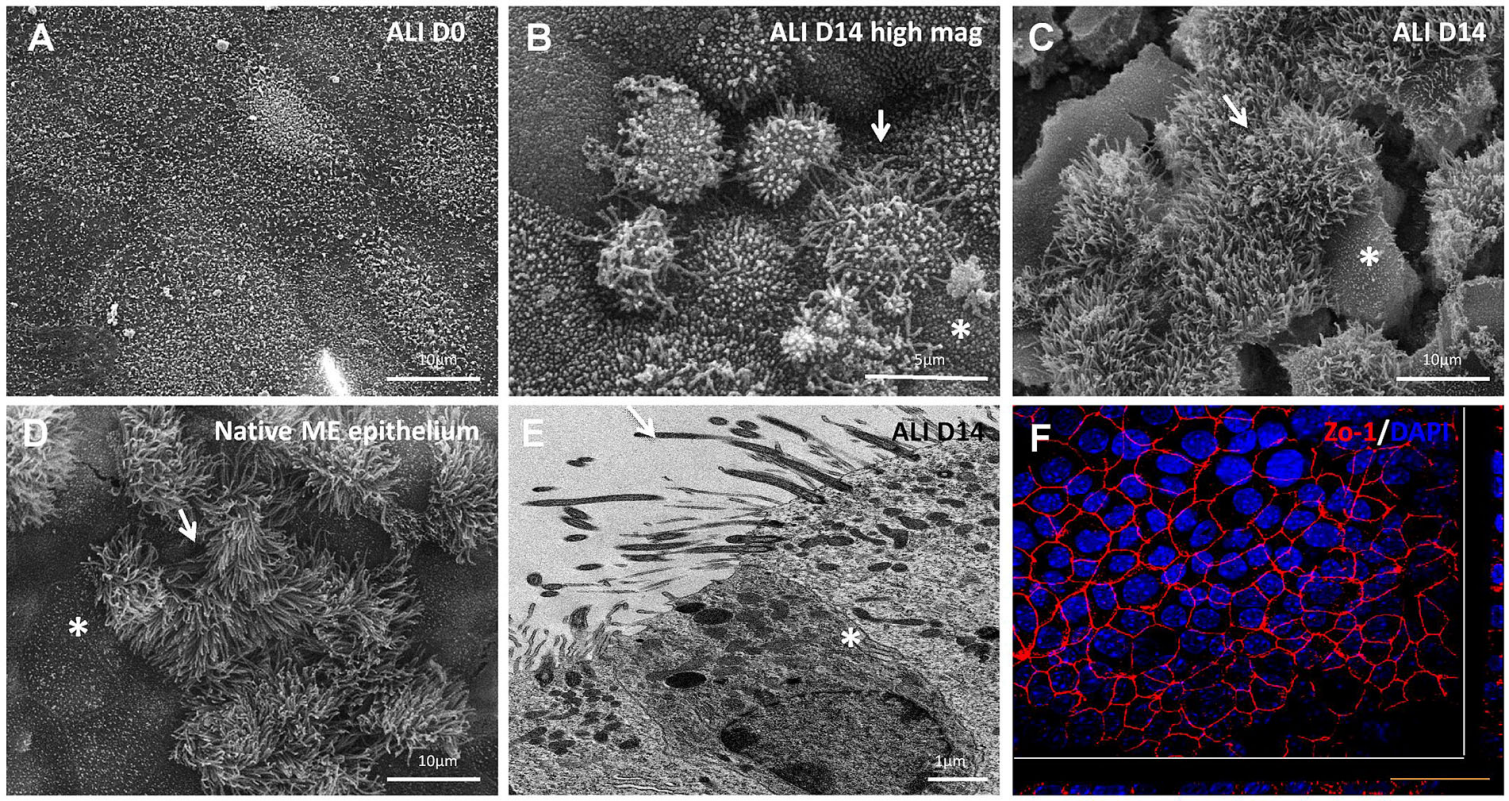
**Electron microscopy of mMEC cultures.** (A-D) Scanning electron microscopy of ALI day 0 mMEC cultures showing large flat polygonal cells with apical microvilli (A), ALI day 14 cultures showing dome shaped cells at higher magnification (B) and combination of interspersed flat polygonal and densely ciliated cell populations a lower magnification (C) resembling the morphology of native middle ear epithelium (D). Cracks in the membrane are due to processing of samples for s.e.m. White arrows mark elevated ciliated cells and asterisks mark flatter polygonal cells. (E) Transmission electron microscopy of ALI day 14 mMEC cultures showing adjacent ciliated and secretory cells and formation of tight junctions demonstrated by presence of desmosomes (asterisk). Arrow shows cilia. (F) Immunofluorescence confocal microscopy image showing formation of tight junctions marked by ZO-1-positive staining. Cross-sections through both axes of the membrane are shown beneath and to the right beyond the white lines. Images are representative of three independent batches. Scale bars: 10 μm in A,C,D; 5 μm in B; 1 μm in E; 50 μm in F.

Electron microscopy analysis during ALI culture revealed the development of a mucociliary epithelium (Fig. 2). At ALI day 0 (undifferentiated cells), scanning electron microscopy showed uniformly flat, large cells with microvilli on the apical surfaces (Fig. 2A). At ALI day 14, the cells exhibited a dome-shaped appearance (Fig. 2B), areas of flatter polygonal and secretory cells with microvilli on the apical surfaces and areas abundant in ciliated cells (Fig. 2C). The morphology of the ALI day 14 mMEC cultures resembled the *in vivo* middle ear epithelium (Fig. 2D). Transmission electron microscopy revealed that ALI day 14 cells were polarised with desmosomes on the basolateral surfaces suggesting the formation of tight junctions, another feature of epithelial cells (Fig. 2E). The formation of tight junctions was further confirmed by uniform expression of ZO-1 in the cell membrane (Fig. 2F).

### Expression of epithelial markers by mMEC cultures

**Fig. 3.**
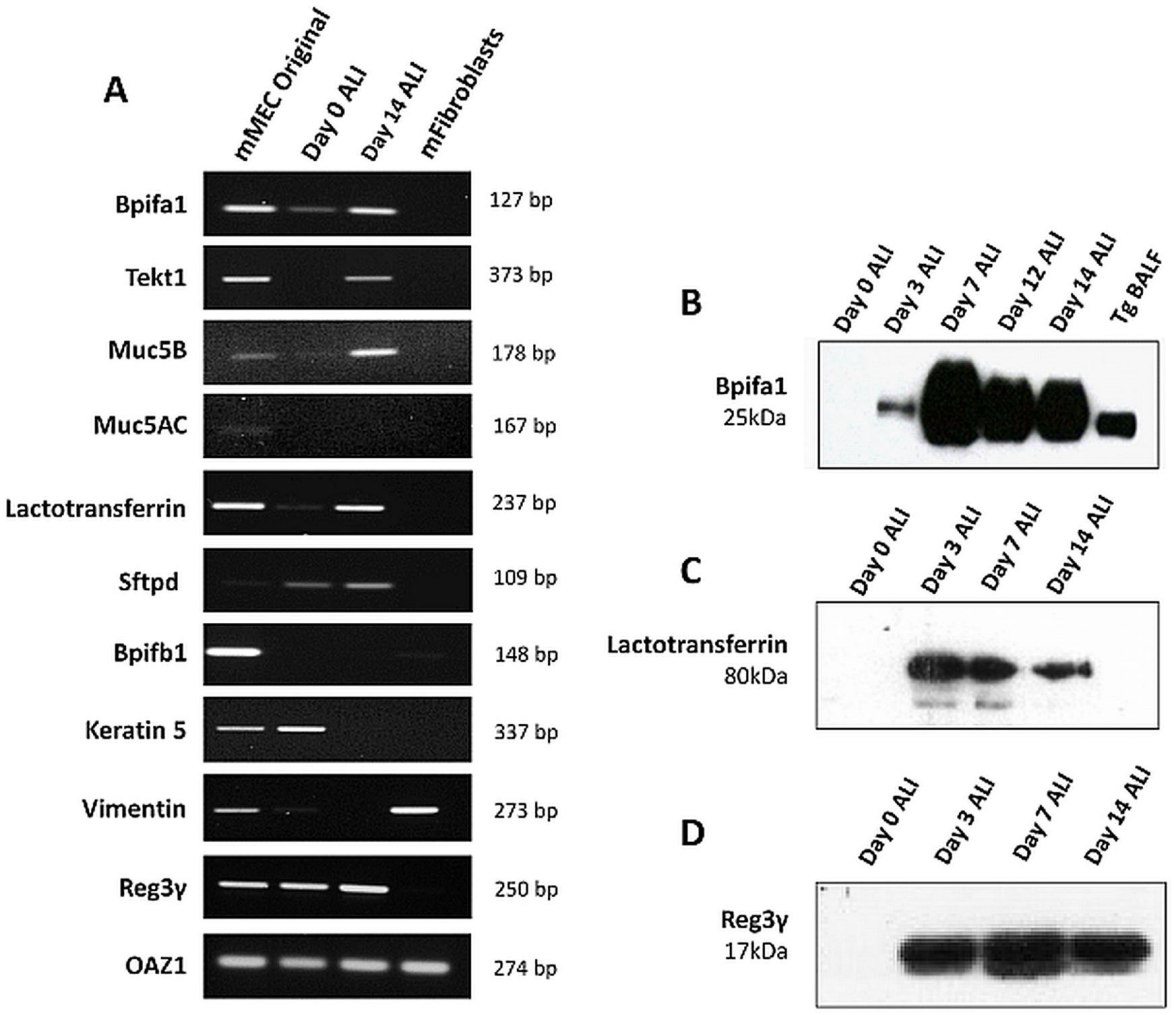
**Expression of epithelial markers in mMEC cultures.** (A) End-point RT-PCR showing expression of a selected panel of upper airway-associated genes in mMEC original cells isolated from the middle ear cavity, ALI day 0 cells and ALI day 14 cells, with fibroblasts as a negative control. Expression profile of ALI day 14 cells was similar to mMEC original cells isolated from the middle ear for most genes. (B-D) Detection of Bpifa1 (B), lactotransferrin (C) and Reg3γ (D) in the apical washes from differentiating cells using western blotting. Data is representative of three independent cultures.

The expression of a selected panel of genes, known to be expressed by the middle ear epithelium and upper airways, was analysed by reverse transcription (RT)-PCR of RNA from the original mMECs before seeding and compared with ALI day 0 and day 14 cells. Fibroblasts isolated by differential adherence were used as a negative control for epithelial markers (Fig. 3A). *Bpifa1* and *Bpifb1* encode secreted, putative innate immune molecules expressed in the upper airways. *Bpifa1* was expressed strongly in the original and ALI day 14 cells, but lower in the undifferentiated ALI day 0 cells. *Bpifb1* was detected only in the original cells, not in cultured cells. *Tekt1* (a marker of ciliated cells) was detected at ALI day 14. Analysis of *Muc5ac* and *Muc5b* expression, markers of goblet cells, suggested that *Muc5ac* was weakly expressed in mMEC original cells but was not detectable in the cultured cells, whereas *Muc5b* was expressed more strongly in the original cells and maintained this expression to ALI day 14. We also studied the mucosal innate immune genes lactotransferrin (*Ltf*), surfactant protein D (*Stfpd*) and regenerating islet-derived protein 3 gamma (*Reg3γ*). Lactotransferrin and *Reg3γ* were detected in the mMEC original cells and at ALI days 0 and 14, whereas expression of *Stfpd* was seen in the cells during ALI differentiation. As expected, the expression of keratin 5, a marker of basal cells, was reduced as cells differentiated from ALI day 0 to day 14. The expression of these epithelial markers in the mMEC cultures indicates that the cells differentiate in culture from ALI day 0 to ALI day 14 and the pattern of expression in the differentiated cells is in line with that seen in the mMEC original cells isolated from the middle ear. The absence of vimentin in ALI day 14 cultures indicates that our mMEC cultures are devoid of fibroblast contamination. *Oaz1* was used as a housekeeping gene for all RT-PCR experiments.

### MS analysis of the apical secretome of mMECs

To complement our gene expression studies, we also performed a global proteomic analysis of apical ALI day 14 secretions of mMEC cells by Orbitrap mass spectrometry (MS). Table 1 lists the most abundant secreted proteins identified classified according to their emPAI score. The most abundant secreted protein was lactotransferrin, with serotransferrin, Reg3γ, lipocalin 2, ceruloplasmin and Bpifa1 also being found at high levels. Multiple anti-proteinase and proteinase proteins were also found in the secretions including members of the WFDC family, WFDC2, WFDC18 (EXPI) and SLPI (WFDC4), as well as multiple cathepsins. We validated the secretion of Bpifa1, lactotransferrin and Reg3γ in the apical washes from the differentiating mMEC cells using western blotting (Fig. 3B-D). The full list of proteins identified is given in Table S1.

**Table 1.**
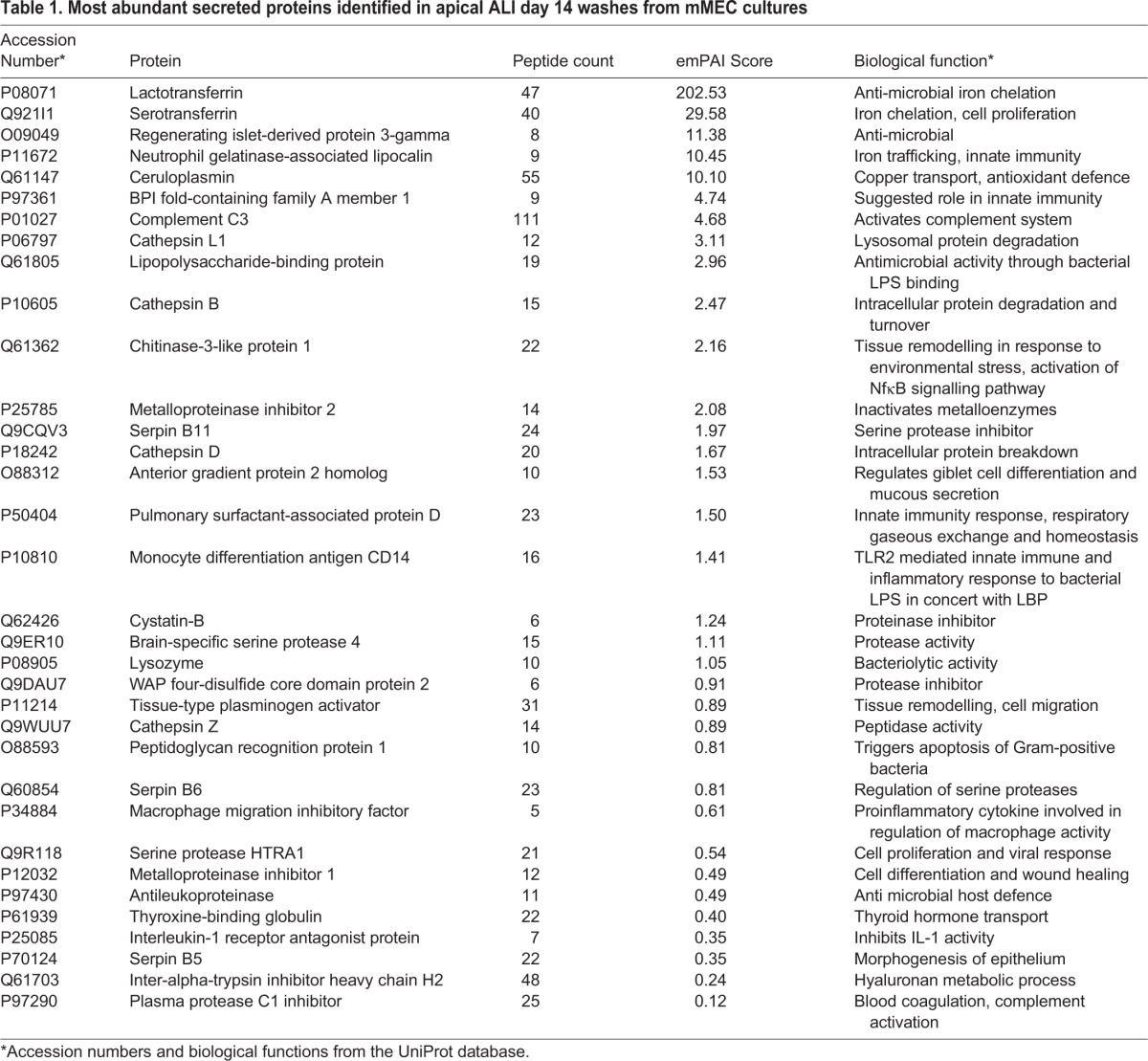
**Most abundant secreted proteins identified in apical ALI day 14 washes from mMEC cultures**

### Localisation of epithelial markers in mMEC cultures

**Fig. 4.**
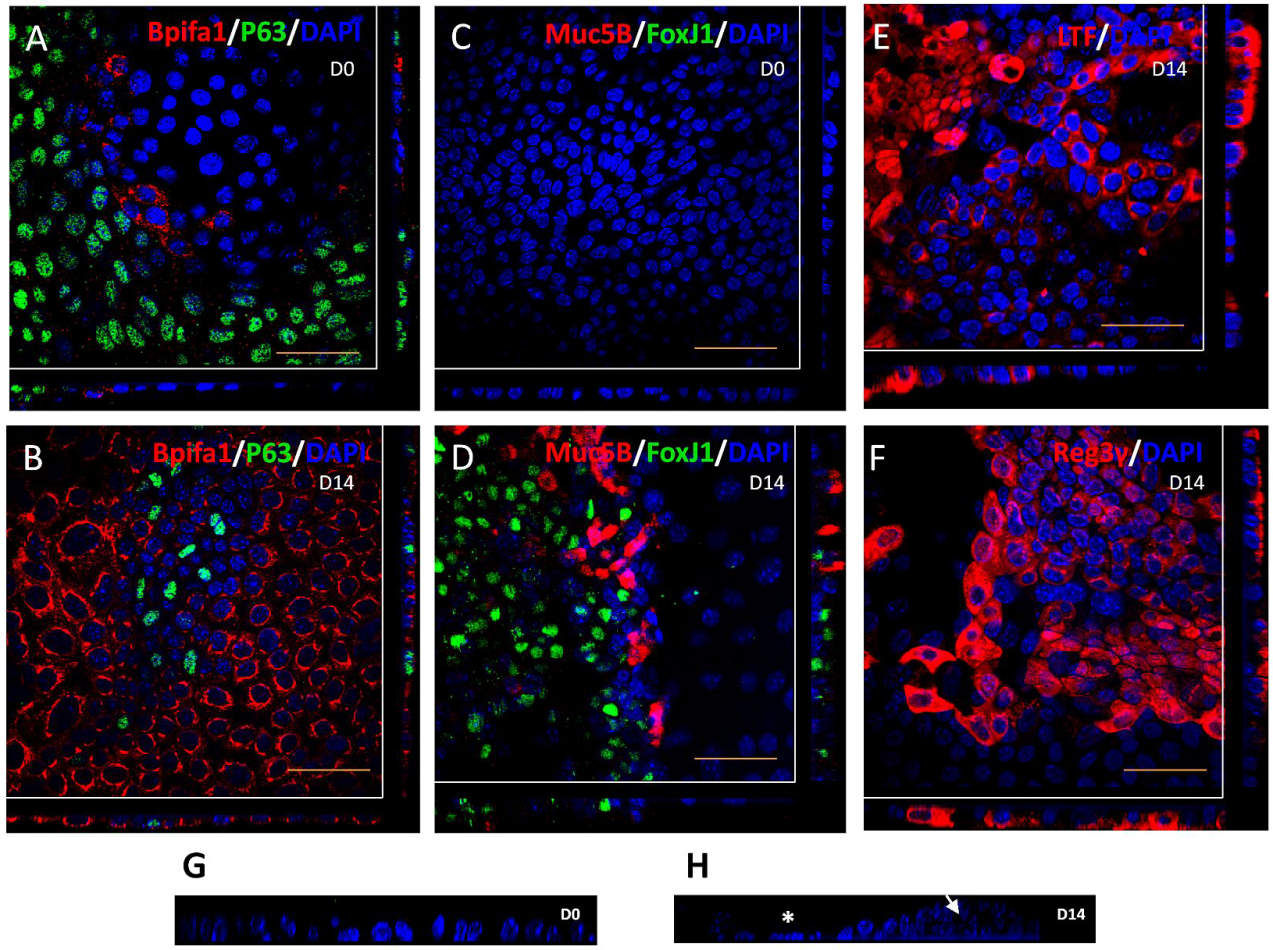
**Localisation of epithelial markers in mMEC cultures.** (A,C) Immunofluorescence confocal images (representative of three independent batches) showing abundant expression of the basal cell marker, P63; limited expression of the secretory protein, Bpifa1 (A), and no expression of goblet cell marker, Muc5B and the ciliated marker, FoxJ1 (C) in undifferentiated ALI day 0 mMEC cultures. (B,D,E,F) Differentiated mMEC ALI day 14 cultures showing expression of secretory cells positive for Bpifa1 (B), lactotransferrin (E), Reg3γ (F), goblet cells positive for Muc5B and ciliated cells positive for FoxJ1 (D). Cross-sections through both axes of the membrane are shown beneath and to the right beyond the white lines. (G,H) High magnification *z*-stack cross sections of nuclei stained with DAPI shows that ALI day 0 cells form a flat monolayer (G) whereas ALI day 14 cells are a combination of pseudostratified elevated cells (arrow) and flatter cells (asterisk) (H). Scale bars: 50 μm.

We used immunofluorescence confocal (IFC) microscopy to study the localisation of epithelial markers in ALI day 0 and ALI day 14 cultures, in order confirm the differentiation process at the protein level. ALI day 0 cultures showed abundant staining of P63 (basal cell marker), scant staining of Bpifa1 (Fig. 4A) and no staining of FoxJ1 (ciliated cells) and Muc5B (goblet cells) (Fig. 4C). However, by ALI day 14, the cells had differentiated into multiple cell types. These stained strongly for Bpifa1, had reduced levels of p63 (Fig. 4B) and were populated with ciliated and goblet cells (Fig. 4D). Bpifa1 was localised the non-ciliated population in differentiated mMECs, consistent with that seen in the *in vivo* middle ear epithelium (Fig. S1). In keeping with proteomic and expression data, we also detected abundant cytosolic levels of lactotransferrin and Reg3γ in the ALI day 14 cultures (Fig. 4E,F). Staining of nuclei with DAPI and *z*-slice imaging using confocal microscopy also demonstrated that the cells formed a flat monolayer at ALI day 0, but showed a more complex reorganisation by ALI day 14 with a combination of flat and elevated cells showing two or three different layers, with nuclei further away from the base of the membrane (Fig. 4G,H).

### Relative abundance of secretory and ciliated cells

**Fig. 5.**
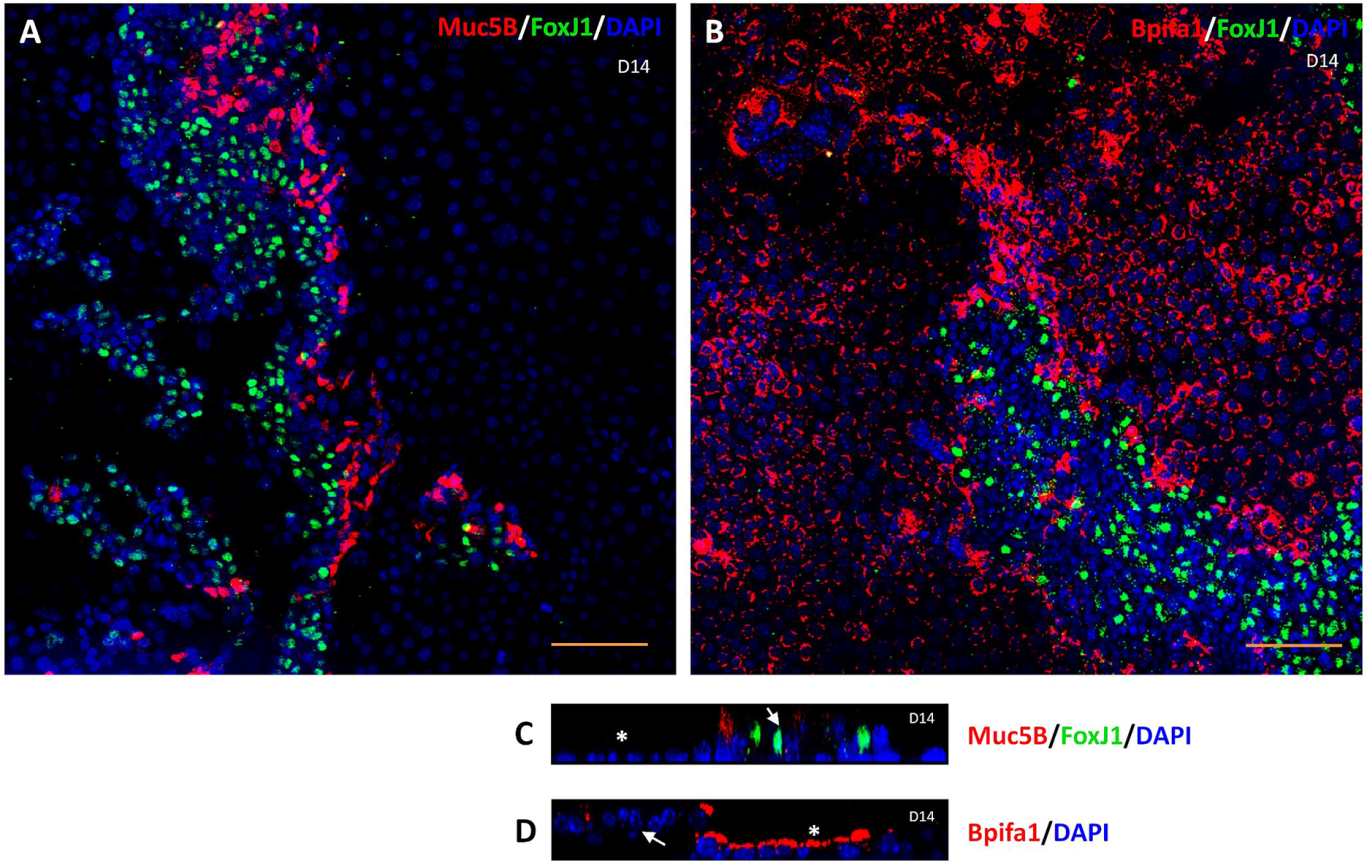
**Distribution of cell types in mMEC cultures.** (A,B) Low-powered (20×) immunofluorescence confocal images representative of three independent batches of ALI day 14 mMEC cultures showing a combination of flat cells and clusters of elevated cells at slightly different foci. Ciliated cells (FoxJ1-positive) do not co-localise with goblet cells (Muc5B-positive) (A) and Bpifa1-expressing cells (B). (C,D) High magnification (60×) cross section *z*-stack confocal images showing that FoxJ1 and Muc5B expression (C) is mostly restricted to the elevated cell types (arrows) and Bpifa1 (D) is predominantly expressed by flatter cells (asterisk). Scale bars: 100 μm in A,B; 50 μm in C,D.

As noted above, phase-contrast microscopy and SEM of mMEC cultures demonstrated that the ALI day 14 cultures were composed of distinct anatomical areas of flat polygonal cells and patches of more elevated pseudostratified cells (Fig. 1H, Fig. 2B). To study this in more detail, we used IFC to determine the localisation of different epithelial markers within the two regions of cellular morphology. FoxJ1-positive ciliated cells and Muc5B-positive goblet cells were restricted to the elevated pseudostratified cell clusters, whereas Bpifa1 was more commonly seen in the flatter cells, although some staining was seen in the elevated cells, especially near the periphery (Fig. 5). This was consistent with our observation that ciliated cells could be seen beating in the elevated clusters of cells under light microscopy. We also confirmed that Bpifa1- and Muc5B-positive cells were not ciliated (Fig. 5A,B). Again, this analysis confirmed the existence of elevated pseudostratified cells within the cultures (Fig. 5C,D).

### mMEC ALI cultures as an otopathogenic infection model

**Fig. 6.**
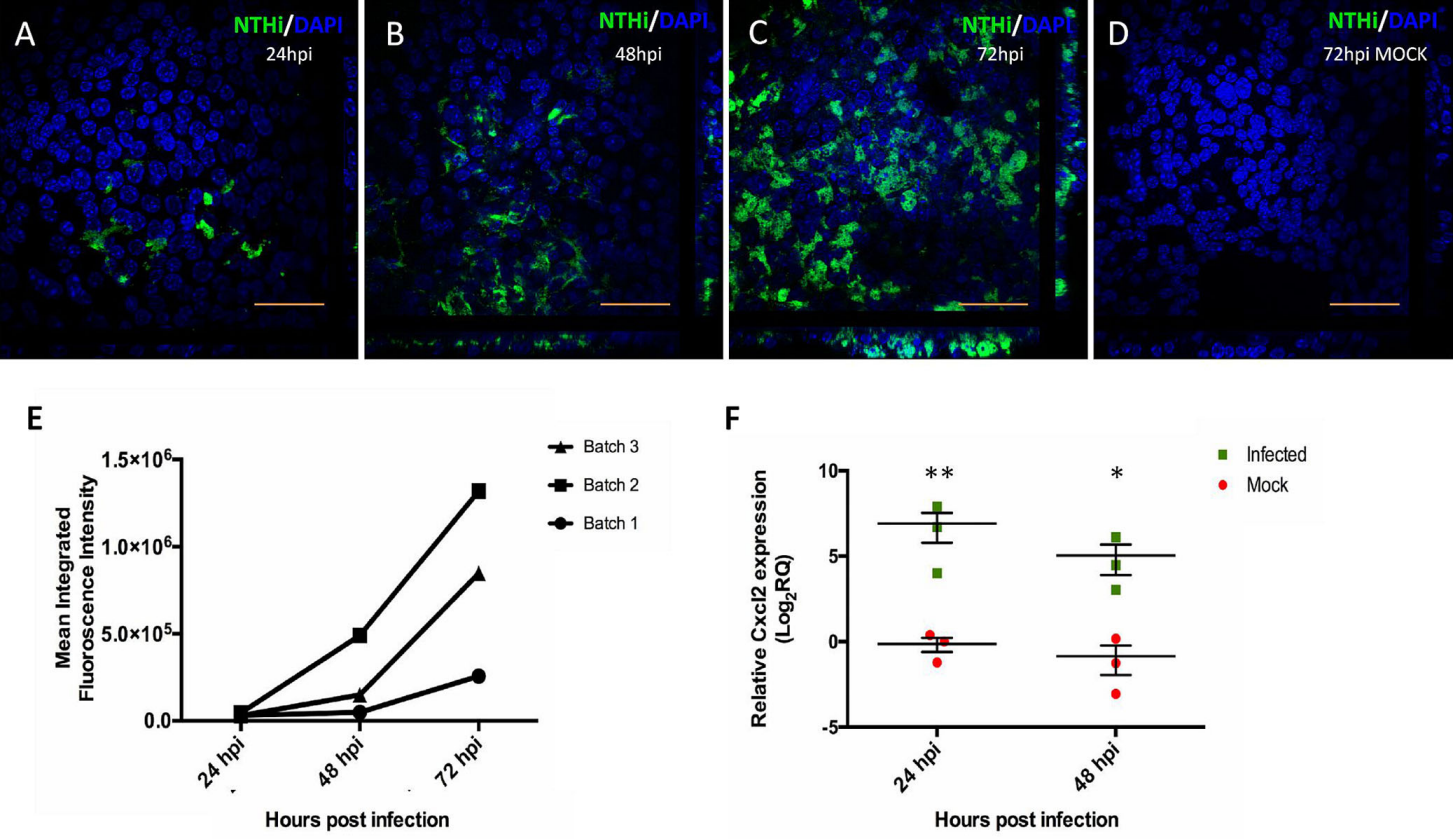
**mMEC cultures serve as an otopathogenic infection model.** (A-C) High magnification (60×) confocal images representative of three independent batches of ALI day 14 mMECs infected with GFP-tagged NTHi-375^SR^ for 24 h (A), 48 h (B) and 72 h (C) showing a time-dependent increase in bacterial infection. (D) Control ALI day 14 mMECs mock infected for 72 h. Scale bars: 50 μm. (E) Increase in mean green fluorescence intensity from 24 hpi to 72 hpi quantified from lower magnification (10×) images from three independent batches, suggesting an increase in the amount of bacteria infecting the cultures with time. (F) Relative gene expression of *Cxcl2* (F) in infected mMEC cultures compared with mock-infected cultures at 24 and 48 hpi. Expression of target gene *Cxcl2* was normalised to three endogenous controls; *ATP5B*, *CyC1* and *Ppia*, and plotted relative to the 24 hpi mock sample. Data was analysed using two tailed Student's *t*-test and represented as mean relative quantification (RQ)±s.e.m. for three independent batches of cultures. *P<0.05, **P<0.01.

Having established a novel mMEC culture model, we wanted to evaluate its utility as a model system for the study of host–pathogen interactions in the middle ear. We infected differentiated ALI day 14 cells with GFP-tagged nontypeable *Haemophilus influenzae* (NTHi) strain NTHI-375^SR^. IFC microscopy indicated that only a few cells were infected 24 hours post-infection (hpi) but by 48 hpi the infection rate had increased and the bacteria continued to spread laterally in culture, infecting the majority of cells by 72 hpi (Fig. 6A-C). We confirmed this observation by quantifying the amount of green fluorescence using image analysis and observed that in every batch the level of bacterial infection increased in a time-dependent manner (Fig. 6E; Fig. S2).

We also studied expression of the pro-inflammatory chemokine *Cxcl2* (*MIP2α*) during the progression of infection. *Cxcl2* was significantly upregulated post-infection at 24 hpi (131-fold) and 48 hpi (36-fold) compared with the 24 hpi mock sample set as the reference (Fig. 6F). This data confirms that our mMEC model is capable of eliciting an inflammatory response.

## DISCUSSION

We have developed a novel *in vitro* model for the culture and differentiation of primary mouse middle ear epithelial cells (mMECs) cultured at an air–liquid interface. The ALI system has previously been used to culture respiratory epithelial cells (TBE) from several species (Clarke et al*.*, 1992; Davidson et al*.*, 2000; Yamaya et al*.*, 1992; You et al*.*, 2002) and more recently, it has been applied to the culture of murine nasal epithelial cells (Woodworth et al*.*, 2007). The exposure of apical cell surfaces to air and the supply of nutrients from the basal compartment mimic the *in vivo* upper airway epithelium and promote differentiation. As the middle ear epithelium can be considered to be an extension of the upper airways and exhibits physiological similarities to the upper respiratory tract, we reasoned that we could extend the use of the ALI system for the successful culture of primary mMECs.

Our mMEC cultures can be used to study both proliferation and differentiation of middle ear cells. The medium used for the culture of mMECs was the same as that used for culture of mouse tracheal epithelial cells, mTECs (You et al*.*, 2002). The media supplements, epidermal growth factor, insulin, transferrin and cholera toxin enhance cell proliferation and ciliogenesis, whereas retinoic acid is important for differentiation of mucous cells (Lechner et al*.*, 1982; Wu et al*.*, 1997; You and Brody, 2013; You et al*.*, 2002). The addition of ROCKi to the culture medium has been shown to enhance basal cell proliferation in airway epithelial cultures (Horani et al*.*, 2013). Using ROCKi, we could maximise the number of transwell cultures established from the limited number of cells isolated from the thin middle ear epithelium without altering differentiation (Fig. S3); an observation that has been made in both human and mouse airway cells (Butler et al*.*, 2016; Horani et al*.*, 2013) and thus reduce the number of animals required for each batch of cells cultured. mMECs grown at ALI differentiated into different cell types by ALI day 14, a typical timescale used for the differentiation of murine respiratory epithelial cells (You et al., 2002). We extended this culture period to ALI day 18 without any morphological differences (data not shown); however, cells cultured to the further time points were not tested.

An important and common problem identified in a number of previous attempts to grow middle ear epithelial cells was the contamination and overgrowth of fibroblasts in the cultures (Nakamura et al*.*, 1991; Tsuchiya et al*.*, 2005; Van Blitterswijk et al*.*, 1986). By adding a differential adherence step, in which fibroblasts adhere to plastic in preference to the epithelial cells, we were able to eliminate fibroblasts from our mMEC cultures, generating a pure epithelial population, as shown by a lack of expression of the fibroblast marker, vimentin, in ALI day 14 cultures.

The mMEC cultures model the native middle ear epithelium as the cells exhibit characteristic epithelial features such as a cobble-stoned morphology, formation of tight junctions, apical–basal polarisation, presence of desmosomes and apical microvilli. The cultures contained a combination of single layers of flatter polygonal cells and clusters of pseudostratified, dome-shaped, elevated cells, with some of these having beating cilia. The dome-shaped appearance of cells can be attributed to an active ion transport mechanism (Chun et al*.*, 2002; Nakamura et al*.*, 1991).

A major limitation of previous middle ear epithelial cultures has been the lack of differentiation into distinct epithelial cell types. The middle ear epithelium, like the upper airway epithelium, is composed of ciliated cells, basal cells, goblet cells and other secretory cells. Previous studies outlined difficulties in maintaining ciliated cells in culture (Nakamura et al*.*, 1991; Ueyama et al*.*, 2001). To our knowledge, the development of ciliated cells has only been described in one study of human middle ear cultures (Choi et al*.*, 2002), also grown at the ALI. Our cultures clearly show the presence of actively beating cilia and also stain for nuclear FoxJ1 protein. We found that the distribution of cilia in our cultures partially mimics that seen in the native middle ear epithelium by SEM (Fig. S4). Parts of the middle ear epithelium are populated with tracts of dense cilia, parts with interspersed ciliated and non-ciliated cells and some parts with a simple epithelium composed of flat non-ciliated polygonal cells. This distribution of cell types within the middle ear cavity is supported by previous studies (Lim, 1979; Thompson and Tucker, 2013) that show that the native epithelium of the hypotympanum and towards the opening of the Eustachian tube (ET) is densely packed with ciliated cells, whereas the mesotympanum contains ciliated islands amongst other non-ciliated cells. The epitympanum is composed primarily of flat squamous cells that might also contribute to the secretory defence system. On this basis, we believe that our mMECs, which are a combination of flat non ciliated, secretory and ciliated cells, closely model the morphology of the native middle ear epithelium.

Mucins are products of goblet cells, unique to the mucosal epithelia, and are essential in the maintenance of mucosal innate defence. A number of attempts have been described to utilise the available middle ear models to study mucin gene expression at the transcriptional level (Kerschner et al*.*, 2010; Liu et al*.*, 2016; Moon et al*.*, 2000; Tsuchiya et al*.*, 2005). However, these studies were limited by the lack of production and localisation of detectable amounts of mucins at the protein level. Goblet cells (as shown by Muc5B positivity) were seen in the elevated pseudostratified clusters of our ALI day 14 mMEC cells in close association with ciliated cells. It has previously been shown that in the native middle ear epithelium the abundance of mucous secreting cells is in parallel to the distribution of ciliated cells, reiterating that our model mimics the *in vivo* middle ear epithelium (Lim et al*.*, 1973). Muc5B is the predominant mucin in COM effusions (Preciado et al*.*, 2010). It has been shown to be indispensable for airway mucocilary clearance and maintenance of mucosal homeostasis and *Muc5b^−/−^* mice develop OM (Roy et al*.*, 2014). Expression of Muc5B at proteomically detectable levels as demonstrated by IFC enables our model to be potentially utilised for further study of the role of mucins in the middle ear epithelium. Moreover, treating primary tracheal and bronchial epithelial cells grown at ALI with IL-13 has been shown to induce goblet cell hyperplasia (Atherton et al*.*, 2003; Kondo et al*.*, 2002). Our model provides a platform to study middle ear mucous hypersecretory phenotypes *in vitro*. The lack of Muc5AC or Muc5B in our MS analysis might be due of the loss of these high molecular weight glycoproteins during the sample processing and preparation.

In addition to ciliated and goblet cells, our cultures also produce a range of other secretory cell products. Bpifa1 is the most widely studied member of the BPI fold (BPIF)-containing family of putative host defence proteins (Bingle et al*.*, 2011) and we have previously shown it to be an abundant product of the murine upper respiratory tract and nasopharynx (Musa et al*.*, 2012). *BPIFA1* was identified as a candidate for OM in a recent GWAS study (Rye et al*.*, 2012) and loss of the protein has been implicated in the development of OM in aged mice (Bartlett et al*.*, 2015). Bpifa1 was readily detectable in mMEC cultures and apical secretions from the cells. Bpifa1 was localised to non-ciliated cells in the cultures, which is consistent with studies from the respiratory tract (Barnes et al*.*, 2008; Kim et al*.*, 2006; Musa et al*.*, 2012). *Bpifb1*, conversely, was detectable in the original cells, but not in the ALI day 14 cultures and was absent from the list of proteins identified in the proteomic analysis performed using mass spectrometry. Bpifb1 protein is localised to goblet cells and minor glands associated with the ET, but its expression is limited in the middle ear epithelium (data not shown). This observation suggests that our mMEC cultures model the middle ear epithelium rather than the ET epithelium.

Proteomic analysis by MS identified a number of secretory proteins in the apical washes from the cells. The most abundant proteins comprised a variety of secretory host defence proteins with anti-microbial roles, proteins involved in cellular proliferation, wound repair, stress response, compliment activity and maintenance of cellular homeostasis. It is notable that our MS data contains a number of proteins also identified in a proteomic analysis of ear exudates from individuals with chronic OM such as lactotransferrin, Bpifa1, lipocalin, lysozyme and various cathespins and complement proteins (Val et al*.*, 2016). Lactotransferrin was the most abundant protein identified in our study. It is an innate immune protein secreted by airway mucosal surfaces that has been shown to play a role in maintenance of middle ear immunity (Bernstein et al*.*, 1974; Lim et al*.*, 2000; Moon et al*.*, 2002). It prevents colonisation of mucosal surfaces by scavenging environmental iron, thus limiting its availability for bacterial growth. Human milk lactotransferrin has been shown to attenuate the pathogenic potential of *Haemophilus influenzae* by proteolytically cleaving two important colonisation factors found on the bacterial surface (Hendrixson et al*.*, 2003) and the administration of apolactoferrin has been shown to reduce bacterial counts in chinchilla middle ears with pneumococcal-induced OM (Schachern et al*.*, 2010). Surfactant protein D was originally identified as a lung surfactant-associated protein but is also expressed in the ME and the ET and has a suggested role in enhancing opsonisation and phagocytosis of bacteria (Lim et al*.*, 2000; van Rozendaal et al*.*, 2001; Wright, 1997). Reg3γ is C-type lectin produced by Paneth cells and secreted into the intestinal lumen with a suggested bactericidal activity against gram-positive bacteria by binding to peptidoglycan in the bacterial cell wall (Cash et al*.*, 2006). It has been shown to spatially regulate the separation of microbiota from the host small intestinal epithelium (Vaishnava et al*.*, 2011). *Reg3γ^−/−^* mice have an altered mucosal distribution and increased inflammatory response in the small intestine (Loonen et al*.*, 2014). *Reg3γ* has also been shown to be involved in pulmonary innate immunity as it is induced by *Stat3* during methicillin-resistant *Staphalococcus aureus* (MRSA) infection in lung and inhibits MRSA growth *in vitro* (Choi et al*.*, 2013). This is the first study reporting the expression of this protein in the ME and it is possible that Reg3γ performs a similar function in the middle ear.

It was noticeable that the proteomic data unexpectedly contained many intracellular proteins. We also detected the presence of the membrane-tethered mucins Muc1, Muc4 and Muc18 (Table S1). This can be reasoned to be the content of secreted exosomes. Exosomes are small membrane-bound units released by the fusion of endosomal micro vesicular bodies with the apical plasma membrane. They have been suggested to be involved in stimulating immune responses, modulating secretory activities and engaging in cell communication by packaging and delivering microRNAs to other cells (Keller et al*.*, 2006; Valadi et al*.*, 2007). Exosomes are released by epithelial cells (Kapsogeorgou et al*.*, 2005; Van Niel et al*.*, 2001) found in bronchoalveolar lavage fluids (Admyre et al*.*, 2003) and also in the apical secretions from human bronchioalveolar epithelial (HBE) cultures (Kesimer et al*.*, 2009; Pillai et al*.*, 2014). The presence of exosome-associated proteins in our mMEC secretome further adds to the potential utility of this model to study the role of exosomes in middle ear biology and OM pathogenesis.

Infection of mMECs using the human otopathogen, NTHi, demonstrates that our culture system can be effectively utilised for the study of host–pathogen interactions within the middle ear. Our studies show that NTHi initially infected a small number of cells in culture and the infection spread laterally over time. Since the advent of pneumococcal vaccines, NTHi is the most common pathogen in OM (Casey and Pichichero, 2004). Epithelial remodelling, one of the most common features of OM, is characterised by mucous metaplasia. The chemokine Cxcl2 is the murine homologue of IL-8 and is a key mediator of overproduction of mucin (Juhn et al*.*, 2008). NTHi infection is known to stimulate *Cxcl2* upregulation in several murine tissues including the middle ear, lungs and the inner ear (Gaschler et al*.*, 2009; Lim et al*.*, 2007; Woo et al*.*, 2012). Moreover, *Cxcl2* was identified as the most upregulated gene when mice were trans-tympanically injected with NTHi and on infection of the mouse middle ear epithelial cell line (mMEC) with NTHi (Preciado et al*.*, 2013). Our data shows that *Cxcl2* was significantly upregulated on NTHi infection at 24 and 48 hpi, suggesting that our mMECs respond to bacterial infection in a manner similar to the native middle ear epithelium. This opens up a new avenue to utilise this system to study the response of middle ear cells to different insults, injuries and infections. Our mMEC cultures can potentially be utilised to study the interaction of host middle ear epithelial cells with a variety of bacterial as well as viral otopathogens.

Previous studies have demonstrated successful gene silencing in primary HBE cells and mTECs using lentiviral transduction systems (Horani et al*.*, 2012; Vladar and Stearns, 2007). Recently the CRISPR/CAS9 genome editing system was utilised in HBE cells and mouse tracheal organoid cultures in order to study genes involved in the regeneration of basal cells into mucociliary cells of the airway epithelium (Gao et al*.*, 2015). The physiological similarities of mMEC cells with airway epithelial cells and the use of similar culture conditions for their growth means that the mMEC model might be amenable to gene editing studies in future.

It is known that primary airway epithelial cells cultured from individuals with cystic fibrosis and asthma maintain the disease phenotype in culture (Davies et al*.*, 2003; Matsui et al*.*, 1998). A number of mouse models are available for the study of OM. These include mice deficient in innate immunity genes such as *Evi1*, *Fbxo11*, *TLRs* and *Myd88*, ciliary development genes such as *Dnahc5* (Rye et al*.*, 2011) and goblet cells (Roy et al*.*, 2014). It will be interesting to see if our mMEC culture system can be utilised to reproduce the OM phenotype of these mouse mutants *in vitro* and enable comparative studies between unaffected and diseased cultures. OM often involves complex responses involving the middle ear epithelium, sub-epithelial mesenchyme, inflammatory cells and middle ear effusion, making it challenging to identify epithelial cell-specific responses. The mMEC culture system will provide us with the ability to isolate and assay responses of specific sub-populations of epithelial cells. Our cell isolation method eliminates the influence of explants and excludes fibroblasts from culture, which were two of the most important confounding factors in previous studies of primary middle ear epithelial cells. It is a three-dimensional model of the middle ear epithelium and hence mimics the *in vivo* physiology more closely than other cell lines. The possibility of replicating the phenotype of the genetic models of OM, the capacity to easily manipulate differentiation of cells by modifying culture conditions and the ability to infect cells with various otopathogens makes our model widely applicable to the wider OM community. The availability of such a well-characterised model of the middle ear epithelium can help better understand the cell biology of the middle ear and improve our understanding of the pathogenesis of OM.

## MATERIALS AND METHODS

### Ethics statement

Humane care and animal procedures were carried out in accordance to the appropriate UK Home Office Project licence. Randomised male and female 8-10-week-old C57BL/6 and C3H/HeH mice, housed in individually ventilated cages (Techniplast UK Ltd) under specific pathogen-free (SPF) conditions were obtained from MRC Harwell, UK.

### Dissection of the middle ear cavity

The detailed protocol used for dissection is outlined in Fig. S5. Mice were euthanised by terminal intra-peritoneal injection of 100 μl pentobarbital (50 mg/ml, Henry Schein) and exsanguinated by cutting the inferior vena cava. Mice were decapitated, the skin at the nape of the neck was incised, bisected anteriorly and removed entirely to expose the bony surface of the skull and the lower jaw was detached under direct visualization. Under a dissecting microscope (Olympus SZx10), the skullcap was gently opened with a pair of fine forceps and the brain was removed. The head was bisected at midline and oriented with the opening of the ear facing upwards. Any muscle, soft tissue and remnant hair surrounding the ear were removed using fine dissecting scissors and forceps, leaving the MEC (bulla), still attached to the outer ear canal (OEC) and the inner ear (IE). The bony shell of the bulla was further cleaned free of any attached extraneous tissue. The OEC, which appears a shade lighter than the MEC, was gently broken away from the MEC using stork­-bill forceps. The tympanic membrane and the ossicles usually detached from the MEC along with the OEC. Alternatively, they were physically removed using fine stork-bill forceps. Lastly, the cup-shaped MEC was carefully lifted away from the inner ear.

### Isolation and differentiation of middle ear epithelial cells at air–liquid interface

The protocol for primary culture and differentiation of mouse middle ear epithelial cells (mMECs) was adapted from a previously described method for mTECs (You and Brody, 2013; You et al*.*, 2002). For each batch of cells, bullae from approximately six mice (12 bullae) were pooled in a tube containing pronase (1.5 mg/ml) in mMEC basic media [DMEM/F-12 HAMs media (Life Technology, 31330-038) supplemented with penicillin (100 µg/ml) and streptomycin (100 µg/ml) (Life Technology, 15070-063)] and subjected to overnight proteolysis at 4°C. The pronase was neutralised by the addition of 10% foetal bovine serum (FBS) and the bullae were gently agitated by inverting the tube 25 times. The bullae were then transferred to 2 ml of fresh mMEC basic 10% FBS media, the tube was inverted again 25 times and this process was repeated three times. The combined proteolytic and mechanical actions led to dissociation of the middle ear cells from the bullae (Fig. S6). Media from the three tubes was combined and centrifuged at 500 ***g*** for 10 min at 10°C. The pelleted cells were re-suspended in 1 ml of media containing 1 mg/ml bovine serum albumin (BSA) and 0.5 mg/ml DNase I (Sigma-Aldrich, DN25). Cell viability and number were assessed using Trypan Blue staining and a haemocytometer. Cells were centrifuged at 500 ***g*** for 5 min at 10°C and the pellet resuspended in 5 ml of mMEC basic 10% FBS media. In order to separate contaminating fibroblasts from epithelial cells a differential adherence step was performed by plating the cells on 60 mm surface-treated tissue culture dishes at 37°C in a 5% CO_2_ incubator for 3-4 h. Fibroblasts attached to the plastic whilst the non-adherent epithelial cells were collected, centrifuged at 500 ***g*** for 5 min at 10°C and resuspended in 1 ml of mMEC plus media [mMEC basic media supplemented with 5% FBS, 30 µg/ml bovine pituitary extract (Life Technology, 13028-014), 10 µg/ml of insulin (Sigma-Aldrich, I1882), 25 ng/ml of mouse epidermal growth factor (BD Biosciences, 354001), 5 µg/ml of transferrin (Sigma-Aldrich, T1147), 0.1 µg/ml of cholera toxin (Sigma-Aldrich, C8052) and 0.01 µM of freshly added retinoic acid (Sigma-Aldrich, R2625)].

For optimisation of culture conditions, cells were plated on either tissue culture plastic or sterile, 0.4 μm pore sized transparent PET (polyethylene terephthalate) membranes coated with 150 μl (50 μg/ml) of rat-tail collagen type I (BD Biosciences, 354236) in a 24-well supported transwell format (Falcon, 353095). mMECs were seeded at an initial density of 1×10^4^ and 2×10^4^ cells/well in the presence or absence of 10 μM of Rho kinase inhibitor, Y-27632 dihydrochloride (ROCKi, Tocris Bioscience, 1254) and 5×10^4^ cells/well without ROCKi. A seeding density of 1×10^4^ cells/well with ROCKi on transwell membranes was identified as optimum and therefore used for culturing all following batches of cells (Fig. S3A-G). An average of 671,788±59,790 (*n*=9 batches) cells were obtained from 12 pooled bullae. 30 to 35 transwells were typically seeded at this density and the remaining cells were lysed in Trizol reagent (Sigma-Aldrich, T2494) to give freshly isolated mMEC original cells for comparison with the cultured cells.

Cells were initially cultured submerged, in mMEC plus (proliferation media) with 300 μl of media in the top chamber and 700 μl in the bottom chamber. Media was changed every 48 h, until the cells were completely confluent, thereafter media from the apical chamber was removed and media in the basal chamber was replaced with mMEC SF (differentiation media) [DMEM/F-12 media supplemented with 1 mg/ml BSA (Life Technology, 31330-038), 5 µg/ml insulin, 30 µg/ml bovine pituitary extract, 5 µg/ml transferrin, 5 ng/ml mouse epidermal growth factor, 0.025 µg/ml cholera toxin and freshly added 0.01 µM retinoic acid] to induce ALI culture. This system of culture promotes differentiation of cells by mimicking the *in vivo* situation. Cells were differentiated at ALI for 14 days and media was changed every 48 h. Cells were lysed in 250 µl of Trizol reagent for RNA extraction and apical washes were collected in 200 µl of sterile HBSS at ALI day 0 (submerged day 10), day 3, day 7 and day 14. Fig. 2A gives a brief overview of the complete cell culture system. Doubling time for cells seeded at 5×10^4^ cells/well in mMEC plus media without ROCKi was determined by trypsinising the cells and using the formula: PD=t×log_2_/(logC2-logC1) where PD, population doubling time; t, time (48 h); log, 10-based log; C1, initial cell count; C2, final cell count.

### Immunoflorescence microscopy

Transwell membranes at ALI day 0 and day 14 were fixed with 10% phosphate-buffered formalin at 37°C for 30 min. Cells were permeabilised using 0.5% Triton X-100. Non-specific binding was blocked using 10% goat serum in PBS and incubation for 1 h at 80 rpm on an orbital shaker at room temperature. The membranes were washed three times with PBS for 5 min at 150 rpm and incubated with the following primary antibodies: anti-Bpifa1 (1:200), anti-Foxj1 (1:300), anti- p63 (1:100), anti-MUC5B (1:100), anti-ZO-1 (1:200), anti-lactotransferrin (1:200) or anti-Reg3γ (1:200), overnight at 4°C at 80 rpm on the shaker. All antibody details are given in Table S2. The following day, membranes were washed three times with PBS for 5 min at 150 rpm and bound primary antibodies detected using 1:200 dilution of the appropriate fluorophore-tagged secondary antibody (Alexa Fluor 568 goat anti-rabbit, A11011 or Alexa Fluor 488 goat anti-mouse, A11001) incubated for 1 h at room temperature at 80 rpm in the dark. Membranes were washed three times as above, detached carefully from their transwell support with a fine scalpel and placed on a glass microscope slide with the cells facing upwards. Nuclei were counterstained using Vectashield DAPI mounting medium (Vector Laboratories) and the cells imaged using an Olympus Fluoview 1000 confocal microscope.

### Scanning electron microscopy

ALI day 0 and day 14 cell membranes were washed free of culture media with sterile, warm HBSS and fixed in 3% glutaraldehyde in 0.1 M sodium cacodylate buffer overnight at 4°C. The membranes were washed twice with 0.1 M cacodylate buffer for 5 min each, detached from their transwell support as described above and post-fixed in 2% aqueous osmium tetroxide. The specimens were washed briefly in water, dehydrated in a graded ethanol series, dried in a 1:1 mixture of 100% ethanol:hexamethyldisilazane (HEX) before final drying in 100% HEX. The membranes were placed overnight in a fume hood, mounted onto a pin-stub using a Leit-C sticky tab, (gold sputter-coated) and examined using a Philips XL-20 SEM at 15 kV.

### Transmission electron microscopy

ALI day 14 cell membranes were fixed with 3% glutaraldehyde in 0.1 M sodium cacodylate buffer, washed and post fixed in 2% osmium tetroxide as mentioned above. The detached membranes were washed briefly in water and dehydrated through a graded ethanol series, cleared in epoxy-propane (EPP) and infiltrated in 1:1 mixture of araldite resin:EPP mixture overnight on a rotor. This mixture was replaced in two changes with fresh araldite resin mixture over an 8-hour period, before being embedded and cured at 60°C in an oven for 48-72 h. Ultrathin sections (∼85 nm thick) were cut on a Leica UC 6 ultra-microtome onto 200 mesh copper grids, stained for 30 min with saturated aqueous uranyl acetate followed by Reynold's lead citrate for 5 min. Sections were examined using a FEI Tecnai transmission electron microscope at an accelerating voltage of 80 kV. Electron micrographs were recorded using a Gatan Orius 1000 digital camera and DigitalMicrograph software.

### Nontypeable *Haemophilus influenzae* infections

mMECs were cultured in antibiotic-free mMEC-SF media for 48 h prior to infection. A GFP-tagged, streptomycin-resistant strain of the clinical OM isolate, NTHi-375 (NTHi-375^SR^), derived from a Finnish pneumococcal vaccine study on children undergoing tympanocentesis in 1994-1995, was used for all bacterial challenge experiments (Cody et al*.*, 2003). NTHi-375^SR^ was grown from glycerol stocks on brain heart infusion (BHI) agar plates supplemented with 2 μg/ml nicotinamide adenine dinucleotide hydrate (NAD; Sigma-Aldrich, N7004), 2 μg/ml hemin (Sigma-Aldrich, H9039) and 200 μg/ml streptomycin sulphate (Melford Laboratories, S0148) at 37°C, 5% CO_2_ overnight. The following day colonies were transferred into BHI broth supplemented with NAD and hemin and incubated for 3 h at 37°C, 5% CO_2_, 250 rpm. The optical density (OD_490_) of 1 ml of liquid culture was spectrophotometrically determined (Jenway 6300) and the culture diluted with PBS to give a concentration of 1×10^9^ bacteria/ml. An appropriate amount of culture in antibiotic-free mMEC SF media was added to the apical chamber of ALI day 14 mMEC cultures such that the membranes were infected at a multiplicity of infection (MOI) of 1:100 (mMECs: bacteria). An equal volume of sterile PBS was added to generate mock-infected controls. The membranes were incubated at 37°C, 5% CO_2_ for 1 h and washed three times with sterile HBSS to remove non-adherent bacteria. Media was replaced in the basal chamber and cultures were incubated for 4, 24, 48 and 72 hpi. At each time point apical washes were collected, cells were lysed in Trizol reagent for RNA extraction and membranes were fixed for IFC as described. Membranes were visualised using confocal microscopy and infection was quantified by measuring the mean integrated fluorescence intensity of four central 10× magnification fields at each time point, in each batch, using ImageJ software (NIH).

### Reverse transcription PCR (RT-PCR)

For end-point RT-PCR, total RNA was extracted from at least three batches of freshly isolated mMEC original cells and mMECs at ALI days 0 and 14 lysed in Trizol. RNA yield was determined using NanoDrop 1000 (Thermo Scientific). Residual genomic DNA was digested by DNase I treatment (Promega, M6101) and 200 ng of RNA was reverse transcribed using AMV Reverse Transcriptase (Promega, M9004). RT-PCR was performed with 1 µl of template cDNA and Maxima Hot Start Green PCR Master Mix (Thermo Fisher Scientific, K1061). The cycling conditions were: 95°C for 5 min; denaturation at 94°C for 1 min (25-35 cycles); annealing at 60°C for 1 min; extension at 72°C for 1 min; final extension at 72°C for 7 min (MJ Research PTC-200). The primer pairs used are described in Table S3. The amplified PCR products were run on a 2% agarose gel containing 0.5 µg/ml ethidium bromide (Dutscher Scientific, 4905006) and bands visualised using a Bio-Rad ChemiDoc XRS+.

### Real time quantitative PCR (RT-qPCR)

For RT-qPCR, total RNA was extracted from at least three independent batches of ALI day 14 cultures infected with NTHi-375^SR^ for 24 and 48 h and their corresponding mock-infected cultures. Residual genomic DNA was removed using the DNA-*free* kit (Life Technologies, AM1906), RNA quantified using NanoDrop 8000 (Thermo Scientific) and integrity checked on an Agilent Bioanalyzer 2100 instrument using RNA 6000 Nano kit. 400 ng of total RNA was reverse transcribed into cDNA in a 20 µl reaction volume using a Superscript III First Strand Synthesis kit (Invitrogen, 11752-050), in accordance with manufacturer's instructions. RT-qPCR was performed using a TaqMan gene expression assay for *Cxcl2* (Applied Biosystems, Mm00436450_m1) on a 7500 Fast Real Time PCR system (Applied Biosystems), with 2× TaqMan Fast Universal Master Mix (Applied Biosystems, 4352042). 10 ng cDNA was added to each reaction and three technical replicates were performed for each assay in each batch. Genetic expression levels of *Cxcl2* were normalised to three endogenous controls; *ATP5B*, *CyC1* (Primerdesign geNorm Reference Gene Selection kit) and *Ppia* (TaqMan, Mm02342429_g1) and analysed using ABI 7500 software v2.0.1 using the 2^−ΔΔCt^ method. Data is presented as mean relative quantification (RQ) and error bars represent standard error of mean (s.e.m.).

### Western blotting

Standard western blotting technique was used to detect secreted proteins in apical HBSS washes collected at ALI days 0, 3, 7 and 14 from the mMEC cultures. An equal volume of 2× SDS loading buffer (20% SDS, 1 M DTT, glycerol, 0.5 M Tris-HCl pH 6.8, 0.2% Bromophenol Blue, protease inhibitors) was added to the wash and proteins denatured at 95°C for 5 min. 40 µl of total sample was resolved on a 12% polyacrylamide gel, transferred to a PVDF membrane using a semi-dry blotting system (Bio-Rad Trans-Blot Turbo) and probed with primary antibodies anti-Bpifa1 (1:200), anti-lactotransferrin (1:2000) and anti-REG3γ (1:5000) overnight at 4°C. The primary antibody was detected using a polyclonal goat anti-rabbit secondary antibody conjugated with HRP (1:2000; Dako, P0448). Primary antibody details are described in Table S2. Protein bands were visualised using the EZ system (Geneflow, 20-500-120).

### Immunohistochemistry

Formalin-fixed, paraffin-embedded serial sections of the mouse head passing through the middle ear cavity were deparaffinised and dewaxed in 100% xylene (Sigma-Aldrich) and rehydrated in 100% ethanol (Fischer Scientific). Endogenous peroxidase activity was blocked using 0.3% H_2_O_2_-methanol (Fischer Scientific). Non-specific binding was blocked by incubating the sections in 100% goat serum for 30 min at room temperature. Sections were washed and incubated in anti-Bpifa1 primary antibody (1:750) overnight in a humidified chamber at 4°C. The following day, sections were washed twice in PBS, incubated in 0.5% biotinylated polyclonal goat anti-rabbit secondary antibody (Vectastain^R^ Elite^R^ ABC kit, PK-6101) for 30 min at room temperature, followed by incubation with the ABC reagent for signal amplification for 30 min. NovaRed mixture (Vector, SK 4800) was used for colour development. Sections were counterstained using Harris's haematoxylin (Thermo Scientific), differentiated in acid alcohol, treated with Scott's tap water, dehydrated in 95% to absolute ethanol, cleared using xylene (Leica, ST 4020), mounted in DPX (Leica Biosystems) and visualised and imaged using a light microscope (Olympus BX61).

### Sample preparation for mass spectrometry

Apical wash secretions from six batches of ALI day 14 mMEC cultures were pooled. TCA precipitation was performed (30% TCA in acetone) at −20°C for 2 h to precipitate soluble proteins (50 µg). Proteins were pelleted at 12,000 ***g*** for 10 min at 4°C and pellets washed three times with ice-cold acetone, air dried and resuspended in 50 mM ammonium bicarbonate, 0.1% RapiGest SF (Waters). Samples were heated at 80°C for 10 min, reduced with 3 mM DTT at 60°C for 10 min, cooled and then alkylated with 9 mM iodoacetamide (Sigma) for 30 min. All steps were performed with intermittent vortexing. Proteomic-grade trypsin (Sigma) was added at a protein:trypsin ratio of 50:1 and incubated at 37°C overnight. The samples were precipitated using 1% TFA at 37°C for 2 h and centrifuged at 12,000 ***g*** for 1 h (4°C) to remove RapiGest SF. The peptide supernatant was desalted using C_18_ reverse-phase stage tips (Thermo Scientific Pierce) according to the manufacturer's instructions, dried and re-suspended in 3% (v/v) acetonitrile, 0.1% (v/v) TFA for analysis by mass spectrometry (MS).

### NanoLC-MS ESI MS/MS analysis

Peptides were analysed by on-line nanoflow liquid chromatography (LC) using the Thermo EASY-nLC 1000 LC system (Thermo Fisher Scientific) coupled with Q-Exactive mass spectrometer (Thermo Fisher Scientific). Samples were loaded onto an Easy-Spray C_18_ column (50 cm, inner diameter 75 µm), fused to a silica nano-electrospray emitter (Thermo Fisher Scientific). Chromatography was performed at 35°C with a buffer system consisting of 0.1% formic acid (buffer A) and 80% acetonitrile in 0.1% formic acid (buffer B). The peptides were separated over a 97 min linear gradient of 3.8-50% buffer B at a flow rate of 300 nl/min. The Q-Exactive was operated in data-dependent mode with dynamic exclusion and survey scans acquired at a resolution of 70,000. The 10 most abundant isotope patterns with charge states +2, +3 and/or +4 from the survey scan were selected with an isolation window of 2.0 Th and fragmented by higher energy collisional dissociation with normalised collision energies of 30. The maximum ion injection times for the survey scan and the MS/MS scans were 250 and 100 ms, respectively, and the ion target value was set to 1E6 for survey scans and 1E4 for the MS/MS scans.

### Protein identification and quantification

Thermo RAW files were imported into Progenesis LC–MS (version 4.1, Nonlinear Dynamics) and only peaks with a charge state between +2 and +7 were picked. Spectral data were exported for peptide identification using the Mascot (version 2.3.02, Matrix Science) search engine. Tandem MS data were searched against translated ORFs from the mouse genome (UniProt release 2015_02; 16,868 sequences; 9,451,355 residues). The search parameters were as follows; precursor mass tolerance was set to 10 ppm and fragment mass tolerance was set to 0.8 or 0.01 Da and two missed tryptic cleavages were permitted. Carbamidomethylation (cysteine) was set as a fixed modification and oxidation (methionine) set as variable modification. Mascot search results were further validated using the machine-learning algorithm Percolator embedded within Mascot. The Mascot decoy database function was utilised and the false discovery rate was <1%, whereas individual percolator ion scores >13 indicated identities or extensive homology (*P*<0.05). Mascot search results were imported into Progenesis LC–MS for relative quantification using non-conflicting peptides.

### Statistics

A paired two tailed Student's *t*-test was used to compare relative Cxcl2 expression between the mock-infected and NTHi-infected samples at each time point. Data was presented using GraphPad Prism version 6.0.
